# Spatiotemporal dynamics of DNA repair proteins following laser microbeam induced DNA damage – When is a DSB not a DSB?^☆^

**DOI:** 10.1016/j.mrgentox.2013.05.006

**Published:** 2013-08-30

**Authors:** Pamela Reynolds, Stanley W. Botchway, Anthony W. Parker, Peter O’Neill

**Affiliations:** a Gray Institute for Radiation Oncology and Biology, Department of Oncology, University of Oxford, Oxford OX3 7DQ, UK; b Central Laser Facility, STFC, Rutherford Appleton Laboratory, Research Complex at Harwell, OX11 0QX, UK

**Keywords:** NIR microbeam, near infra-red multiphoton laser microbeam, USX, ultrasoft X-rays, ROS, reactive oxygen species, GFP, green fluorescent protein, YFP, yellow fluorescent protein, RFP, red fluorescent protein, DNA damage, DNA repair, Laser, NHEJ, BER

## Abstract

The formation of DNA lesions poses a constant threat to cellular stability. Repair of endogenously and exogenously produced lesions has therefore been extensively studied, although the spatiotemporal dynamics of the repair processes has yet to be fully understood. One of the most recent advances to study the kinetics of DNA repair has been the development of laser microbeams to induce and visualize recruitment and loss of repair proteins to base damage in live mammalian cells. However, a number of studies have produced contradictory results that are likely caused by the different laser systems used reflecting in part the wavelength dependence of the damage induced. Additionally, the repair kinetics of laser microbeam induced DNA lesions have generally lacked consideration of the structural and chemical complexity of the DNA damage sites, which are known to greatly influence their reparability. In this review, we highlight the key considerations when embarking on laser microbeam experiments and interpreting the real time data from laser microbeam irradiations. We compare the repair kinetics from live cell imaging with biochemical and direct quantitative cellular measurements for DNA repair.

## 1 Introduction

The cellular response to DNA damage is vital to maintain genome integrity and stability. It has been postulated that as many as 50,000 lesions are formed daily within the mammalian genome by endogenous damaging agents, such as reactive oxygen species (ROS) produced during cellular metabolism [1]. Mammalian cells are also exposed to exogenous DNA damage from ultra violet (UV) light, ionizing radiation (IR) and radiomimetic agents, to name but a few.

In mammalian cells, a number of DNA repair pathways are utilized to respond to the plethora of lesions induced within cellular DNA through endogenous or exogenous processes. Failure of these pathways to repair faithfully may lead to replication stress, mutations, genetic disorders and cancer. Using sparsely ionizing (low linear energy transfer [LET] radiation (IR)), such as gamma radiation and X-rays, or densely ionizing (high LET) radiation, such as alpha particles and heavy ions, the repair of simple and structural/chemical complex DNA damage sites has been examined generally using immuno-histochemical approaches. For instance ionizing radiation induced foci (IRIF) have been used extensively to investigate the repair of DNA damage. IRIF investigations in fixed cells, however, yield little information on the spatiotemporal dynamics of DNA repair proteins at the damage sites as each time point is based on a different population of cells.

Although many of the key proteins involved in DNA repair have been identified using immuno-histochemical approaches and in part characterized by other *in vitro* and *in vivo* studies, these approaches do not generally provide information on the kinetics of recruitment of proteins to and repair of DNA damage in real time, in living mammalian cells. In recent years, heavy ion beams and laser microbeam techniques have been used to induce highly localized DNA damage in living cells [2–12] to determine the spatiotemporal aspects of DNA damage repair at early times. In this review we have focused on non-homologous end joining (NHEJ) or base excision repair (BER).

Some of the initial experiments investigated the repair of 6-4-photoproducts and cyclobutane pyrimidine dimers induced within a few minutes by UV lasers [2,8,11,13]. In addition, UVA lasers (315–400 nm) have been used in combination with photosensitizers to induce oxidized DNA base damage and single strand breaks (SSBs) [2,8–12]. More recently, near infra-red multi-photon laser microbeam systems (NIR microbeam) have been developed to induce DNA damage in a femto-litre volume of the nucleus, allowing three dimensional resolution in living mammalian cells [14–19] without significant heating effects [18]. NIR microbeam irradiation has allowed detection of repair proteins at sites of DNA damage at early times post irradiation. In contrast real time studies on recruitment of proteins to damage induced by sparsely ionizing radiation have previously been difficult to undertake due to the limitations of observation of foci following broad field irradiation of the cells, although more recently tracks of foci have been detected in real time in living cells using high LET ion microbeams [7] or ultrasoft X-rays (USX) through a shielded grid [3,20–22]. The studies on live imaging of proteins in cells have generally used proteins with fluorescent tags, namely green, yellow or red fluorescent proteins (GFP, YFP, or RFP respectively) as examples, to investigate the real time recruitment, interactions and loss at early times post irradiation at sites of DNA damage. Additionally, laser microbeams can be used to target subcellular regions such as heterochromatin and euchromatin in DNA to determine the impact of chromatin state in real time on DNA repair kinetics.

The aim of this review is to highlight some of the conflicting results observed following laser microbeam irradiations using different wavelengths of laser light when investigating the spatiotemporal effects of DNA damage repair processes. The complications of comparing findings from laser micro-irradiation with light of different wavelengths have previously been discussed when using fixed cells and immuno-histochemistry [2]. The controversy is not helped by the often lack of details of the laser parameters and the methods used in reports. We encourage vigilance in this regard by the reviewers as well as characterization of the temporal width and laser spot profiles of the femtosecond laser pulses that are ultimately delivered onto the sample following transmission through the microscope optics to permit absolute peak powers to be calculated. Additionally, the profile of the types of DNA lesions induced by laser microbeams significantly depends on the wavelength of the radiation [2,23–26]. In this review, we will focus on how the differing profile of lesions on laser wavelength may influence key proteins involved in NHEJ and BER and stress some of the differences observed using alternative laser microbeam set-ups and the kinetic data obtained from real time studies on living cells.

## 2 DNA lesions induced following IR and laser microbeam irradiation

IR induces mainly DNA base modifications, SSBs and double strand breaks (DSBs) in mammalian cells in readily quantifiable yields, expressed as the number of lesions/cell/Gy. For instance, in mammalian cells, IR induces around 850 pyrimidine lesions, 450 purine lesions, 1000 SSBs and 20–40 DSB/cell/Gy with gamma radiation (Table 1, data used from [27–29]) whereas photo-products are not formed. In addition, due to the spatial distribution of lesions induced by low LET radiation tracks, not only isolated lesions but also damage sites of varying structural and chemical complexity containing two or more lesions are also formed, representing ∼30% of the energy deposited. This value increases to 90% for densely ionizing radiation [30]. These structural and chemical complex damage sites have reduced reparability when compared to that of individual lesions [31–33].

In contrast to the knowledge of the types of damage and their quantitation for IR, the profile of lesions induced by laser microbeams depends on the wavelength of the radiation [2,22–25]. At wavelengths between 290 and 340 nm, mainly photo-products are formed together with low yields of SSBs and lesions excised by the glycosylase Fpg, which generally excises oxidized purines [23]. At wavelength >340 nm, the yield of photo-products continues to decrease reaching a background level at ∼420 nm whereas the yield of Fpg sensitive lesions increases reaching a maximum between 400 and 450 nm where a few SSBs are also induced [21]. With laser irradiation, it should also be noted that the density of lesions is dependent on the laser power (*Note*: for pulsed lasers used in microbeams the laser power refers to peak power per pulse and not the average power) used and the dwell time of the laser during irradiation. In contrast to broad field, low LET irradiation, which produces homogenously distributed DNA damage, laser microbeams and more recently USX [3,21,22] and heavy ion beams (reviewed in [34]) are becoming valuable tools for studying the repair of DNA lesions that are induced as tracks (stripes) of DNA damage in living cells. In particular, laser microbeam irradiation has been used to study the real time kinetics of recruitment and loss of fluorescently tagged proteins at damage sites. The recruitment of a variety of repair proteins to sites of DNA damage induced has allowed a number of lesions induced by laser microbeam irradiation to be inferred but not generally verified by alternative methods. This problem in part reflects the high powers used with lasers so that the density of damage is in the range of high LET charge particles as suggested by Splinter et al. [35] for UV laser irradiations and discussed later.

A number of studies using laser microbeams also utilize DNA photosensitizers such as bromodeoxyuridine (BrdU) and Hoechst 33258 (absorbing maximally at 340–355 nm) to induce DNA damage indirectly [2,3,11,36–38]. DNA photosensitizers induce numerous lesions within the DNA including base lesions, SSBs and DSBs (Table 1) [2,3,11,36–39] at the same time minimizing the contribution of UV-type damage. Not only the presence of photosensitisers but also the laser power may greatly influence the findings [40] as discussed below. The caveat of using photosensitizers is that the repair of DNA lesions may be hindered by the presence of the DNA interchelator and the exact mode of action of the photosensitizers, following excitation, electron transfer etc., has yet to be fully characterized.

NIR multi-photon microbeam irradiation has advanced the studies of DNA repair kinetics by minimizing damage to within a femto-litre volume of the nucleus, typically across a 300 nm focal laser spot sufficiently intense to drive the multiphoton process or slightly larger ∼1 μm for single photon UV radiation studies (e.g. 365 nm), although this technique also induces a broad range of DNA lesions, that are dependent on both the wavelength and laser power used. The mechanism of DNA damage induction by NIR multiphoton excitation can occur by 3-photon absorption to directly excite electronic states or indirectly by the production of ROS in close proximity to the DNA, particularly by 2-photon absorption when a photosensitizer is present [15,16,41–43]. Following 3-photon excitation, UV lesions [2,11,13], base lesions [2], SSBs [14,43] and DSBs [4,43] are induced within the DNA (Table 1). Although NIR microbeam irradiation has been a useful tool for investigating the repair of DNA lesions at early times (<1 min) following damage initiation, thus far it has been difficult to determine the yields of DNA lesions produced within the irradiated area. Harper et al. [39] estimated the yields of DSBs, from comparison with the yields induced using USX and a shielded grid system together with γH2AX, to be equivalent to irradiation with 2 Gy (equivalent to 40 DSBs) whereas Bekker-Jensen et al. [36], using either p53 phosphorylation or RPA fluorescence, estimated the yield to be equivalent to 3–10 Gy (equivalent to ∼60–200 DSBs) of low LET radiation using the respective NIR microbeam set-ups. The estimate in Ref. [36] was questioned by the findings of Splinter et al. [35] indicating that the levels of DNA damage are equivalent to several hundred Gray at the same laser wavelength and power settings as those in [36]. Additionally, they proposed that the damage potential of UV laser irradiation is in the range of high LET charged particles. Uematsu et al. [5] and Kleppa et al. [44] estimated the yield of DSBs to be 1000–1500 for 800 nm multiphoton system whereas the 365 nm laser used in their system produces ∼3000 DSBs. Table 1 broadly summarizes the different types of lesions induced by the different radiation types and wavelengths and only with IR is quantification of the yields of the various types of DNA damage presently adequate from knowledge of the dose delivered.

In mono- and multi-photonic studies it has generally been assumed that damage induced within the irradiated area are isolated lesions. Complex damage sites, where two or more lesions are formed close to each other particularly at higher laser powers, have not generally been considered, even though potential differences in the repair pathways and the repair kinetics of the damage may be apparent. For instance, NIR microbeam irradiation has recently been shown to produce a high proportion of complex DNA damage with considerably longer repair times than those for isolated lesions [3,39]. Additionally, a simple DSB when repaired by NHEJ may utilize a different repair sub-pathway of NHEJ for structurally and chemically complex DNA DSBs [3]. Careful consideration of the types of DNA lesions and their density is required when examining the repair kinetics of DNA damage following laser irradiation, as the spectrum of damage may range from simple, isolated lesions to more complex DNA damage, which potentially require different sub-sets of repair proteins and pathways. Use of DNA intercalating photosensitizers may also influence the density of lesions and the relative complexity of damage sites formed relative to that in the absence of the sensitizer, due to the site specific absorption of light and formation of ROS in the DNA by the DNA intercalating sensitizer.

## 3 Repair kinetics of NIR laser microbeam-induced DNA damage

Before discussing repair of DNA damage induced by laser microbeam irradiation, we will initially give a brief background to the issues relating to broad field irradiation with low LET IR. Several of the key proteins involved in DNA repair/signaling following IR have been investigated through co-localization of foci at damage sites detected using immuno-histochemical approaches. The most commonly used is phosphorylation of H2AX, γH2AX, as a DSB marker since the foci develop over megabase pairs from the DSB with many H2AX molecules become phosphorylated within 20–30 min to form visible radiation-induced foci (RIF). Ease of observation of these foci is also dictated through the specificity of the antibody for the phosphorylated form of H2AX so that minimal background signal will interfere. In contrast, for many DNA repair proteins (e.g. Ku70/80), only one or two molecules [45–47] are recruited to sites of DNA damage, such as DSBs, during their repair and therefore do not form readily visible RIF, even following immuno-staining. With low LET IR, the homogeneous distribution of induced damage within the nucleus makes it difficult to visualize one to two molecules of fluorescently tagged repair proteins as foci above the background fluorescence levels. In addition, real time visualization of DNA repair proteins at early times following damage induction is difficult with low LET as irradiation times tend to take a few minutes, i.e. at low dose rate, requiring the cells to be kept at low temperature to minimize damage repair during irradiation. Heavy ion beams have been used for real time kinetics, as they produce distinct tracks of damage within the nucleus [7], similar to laser microbeam systems as discussed in Section 3.2. These sources of high LET radiation tend to provide information on the dynamics of repair of structurally and chemically complex damage sites, therefore limiting its use as a direct comparison to the majority of damage induced by low LET IR.

### 3.1 Real time recruitment and loss of the NHEJ proteins, e.g. Ku80 and DNA-PKcs, at sites of laser damage in living cells

As discussed in Section 2, the DNA damage profile produced following laser microbeam irradiation is wavelength dependent and distinct from that produced by low LET IR. In addition, the density of the lesions induced in the ‘laser track’ is highly dependent on the laser power and at high powers may in part simulate complex damage sites induced by high LET radiation tracks as discussed earlier. Consideration of the laser set-up is particularly important for NIR microbeam experiments where the multiphoton process is highly dependent on the peak intensity at the focal spot [18,19]. To date, the different set-ups for laser microbeam irradiation have not been standardized [2–6] so that variations in intensity may lead to differences in the kinetics for recruitment and loss of repair proteins during DNA damage repair, reflecting different densities of lesions within the ‘laser track’. Several studies have reported on the real time kinetics for recruitment and loss of proteins involved in NHEJ repair of DSBs such as Ku80-EGFP and DNA-PKcs-YFP [2–5,40], where only a few molecules are recruited. However, other studies have failed to visualize these proteins along the laser-induced damage track using antibody staining [36]. Bekker-Jensen et al. [36] suggested that Ku80-EGFP is only visualized at high laser powers (*λ* = 337 nm) when the BrdU-containing cells would no longer be viable. Recently, the recruitment of Ku80 in real time was barely seen at damage sites when using low NIR laser powers at 800 nm, in contrast to their observation when high powers were used [44] as previously described [4,40]. An alternative suggestion, similarly suggested by Kleppa et al. [44], is that the main types of damage sites induced at lower powers in the above studies [36,44] are not DSBs, unless an appropriate photosensitizing dye is present [40]. However, DSBs may arise at the higher powers due to the increased density of lesions so that an increased probability of two bistranded SSBs being formed within <10 bp, the distance separation for two SSBs to give a DSB, as the power is increased.

**Fig. 1:**
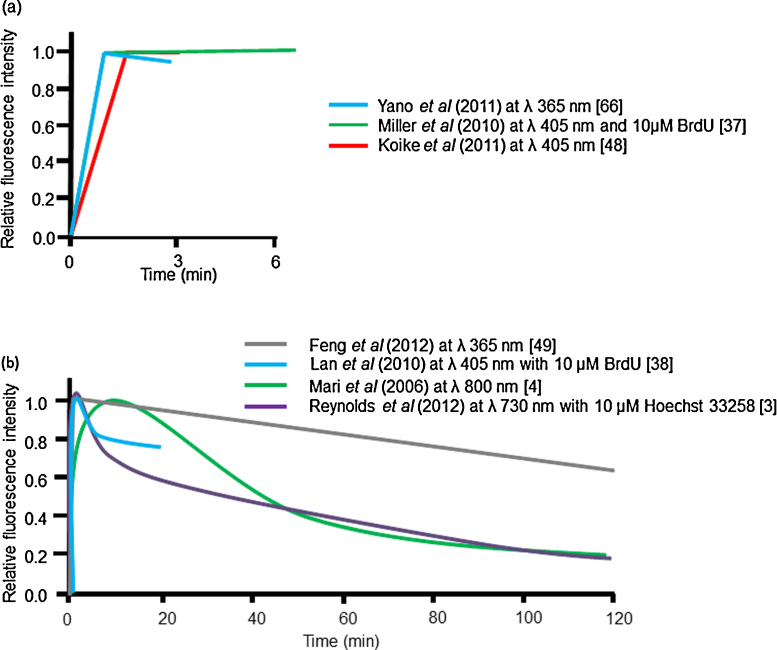
The real time kinetics of Ku80-EGFP/Ku70-GFP at sites of laser microbeam induced DNA damage. The schematic diagrams summarize the data from experimental studies using alternative laser set-ups in the presence and absence of DNA photosensitizers. (a) Represents Ku80-EGFP kinetics in studies that have visualized Ku80-EGFP [48,66] or Ku70-GFP [37] for up to 6 min and (b) represents Ku80-EGFP kinetics from studies that have visualized Ku80-EGFP up to 2 h post irradiation [3,4,38,49].

Several other studies using different laser wavelengths have shown that Ku80-EGFP is rapidly recruited to laser-induced DNA damage (Fig. 1) but the persistence of Ku80-EGFP at these sites is very dependent on the excitation wavelength of the laser. For instance, with 405 nm laser irradiation in the absence (Fig. 1a) or presence (Fig. 1a and b) of BrdU, Ku80-EGFP rapidly accumulates at sites of induced DNA damage and persists for >5 min post irradiation [37,38,48]. Similarly with 365 nm laser irradiation, a rapid accumulation of Ku80-EGFP (Fig. 1a and b) was seen followed by a loss of ∼40% Ku80 fluorescence by 120 min (Fig. 1b). In contrast, following NIR microbeam irradiation at 730 nm in the presence of Hoechst dye [3] or 800 nm at high power but in the absence of dye [4] the half-life of Ku80-EGFP at sites of damage is 40–75 min. This loss is significantly faster than that seen following 365 laser irradiation [49], although a rapid loss of Ku with a half-life of ∼2 min, assigned to simple DSBs, was reported for ∼30% of the damage sites by Reynolds et al. [3] using NIR microbeam irradiation at 730 nm. These kinetic observations [3] are independent of the phase of the cell cycle. The addition of DNA photosensitizers is also shown to alter the kinetics of repair (Fig. 1a and b). This may be due to a change in the chemistry occurring at the damage site when the photosensitizers are excited leading to the formation of different lesions when compared to those formed in the absence of photosensitizers, although one cannot rule out an influence, in part, of the different cell types used.

**Fig. 2:**
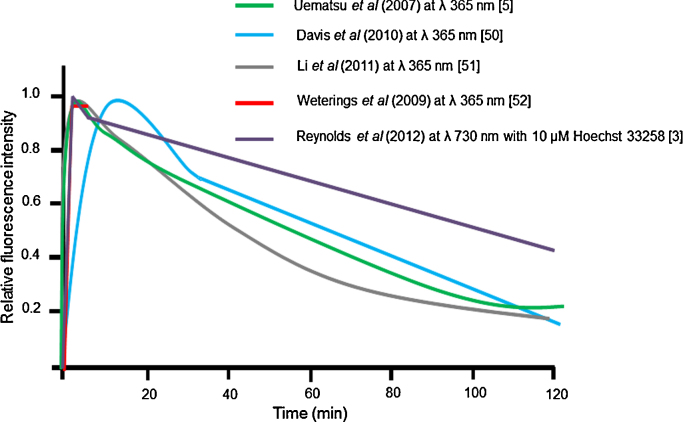
The real time kinetics of DNA-PKcs-YFP at sites of laser microbeam induced DNA damage. The schematic diagrams summarize the data from experimental studies using alternative laser set-ups in the presence and absence of DNA photosensitizers [3,5,50–52].

In contrast to the variations in the kinetics and the relative proportions of slow and fast decaying components seen with Ku80 using different laser wavelengths, the findings on the kinetics for recruitment and loss of fluorescently-tagged DNA-PKcs at damage sites induced by laser microbeam irradiation tend to be similar [3,5,50–52]. For instance, the kinetics for loss of DNA-PKcs following irradiation with either 365 nm lasers or NIR laser at 730 nm occur with mono-exponential decay kinetics (Fig. 2) with similar half-lives of ∼70–100 min. This similarity in the kinetics following laser microbeam irradiation at either 365 nm or 730 nm probably reflects the complexity of the damage induced by both laser set-ups, especially at the high powers used (see Section 2). The high density of laser-induced damage is consistent with the notion that DNA-PKcs is mainly involved in the repair of structurally and chemically complex DSBs [3]. Additionally, it has been shown that DNA-PKcs is involved in the repair of complex damage produced by heavy ion beam irradiation [53,54]. Potential variations in the complexity of the damage induced by alternative laser microbeam set-ups may not have such a big influence on the kinetics of repair as that seen when proteins are involved in the repair of simple DSBs that are easily repaired. The ease of repair of simple DSBs is exemplified when using USX which induces mainly simple DSB [3].

Several of the findings on the persistence of Ku80 or DNA-PKcs at sites of damage are also inconsistent with the lifetime of DSBs induced in cells by low LET IR and measured by pulsed field gel electrophoresis (PFGE) or the comet assay. Using these assays to determine DSB rejoining kinetics, ∼50–60% of DSBs induced by USX are repaired within 30 min [41,55–57]. This level of repair is similar to the level of Ku80 and XRCC4 loss within 30 min from sites of damage induced by USX [3], where the majority of DSBs induced are simple. Additionally, from the loss of Ku80 from and the rejoining of DSBs induced by USX, it is inferred that the majority of DSBs are repaired before the levels of γH2AX, a marker of DSBs, have reached a maximum (within 20–30 min for low LET radiation), highlighting potential difficulties in quantification of induced damage levels based on γH2AX foci. The timescale for loss of DNA-PKcs from damage sites induced by different laser systems as discussed above is similar to that for rejoining of DSBs induced by high LET radiation [29,55], when the majority of the DSBs are thought to be complex [58].

### 3.2 Real time recruitment and loss of BER proteins at sites of laser damage in living cells

**Fig. 3:**
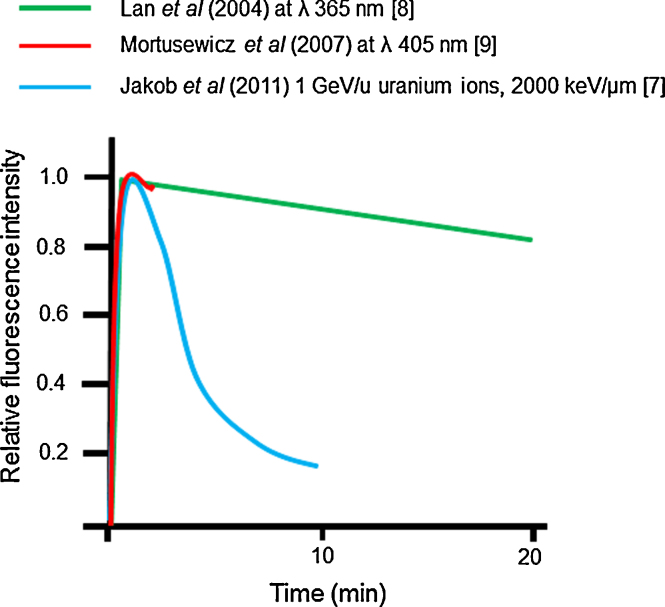
The real time kinetics of XRCC1-YFP at sites of laser microbeam and uranium ion induced DNA damage. The schematic diagrams summarize the data from experimental studies using alternative laser set-ups and heavy ions in the absence of DNA photosensitizers [7–9].

Recent studies using laser microbeams have investigated the kinetics of repair of base lesions and SSBs by BER. A number of fluorescently-tagged proteins involved in the repair of base lesions and SSBs have been shown to be recruited to laser microbeam induced damage. If we concentrate on XRCC1 (a key protein involved in BER), different dynamics have been reported for its recruitment and retention at sites of laser induced damage, reflecting differences in the wavelength of the laser light used. Following 365 nm and 405 nm laser irradiation [8,9], XRCC1 persists at damage sites (Fig. 3). This persistence is inconsistent with the loss of XRCC1 with a *t*_1/2_ ∼ 3 min from damage sites induced by irradiation with uranium ion particles (Fig. 3) [7]. The rapid loss of XRCC1 from DNA damage seen following tangential uranium ion irradiation is consistent with the rapid loss of a proportion of SSBs measured by alkaline elution [59]. To explain these differences, it is tempting to speculate that the damage produced by 365 nm and 405 nm laser light is highly complex, possibly reflecting a high density of lesions induced, and as a consequence the reparability of these complex DNA damage sites is reduced, leading to persistence of XRCC1 at the damage sites. Furthermore it is known that complex damage sites, also known as clustered DNA damage sites which contain two or more lesions within one or two turns of the DNA, are difficult to repair [60–63]. The differences seen only highlight the need for essential details of the laser irradiation settings to be given for better comparison of the findings.

## 4 Conclusion

Laser microbeams are a valuable tool for studying the spatiotemporal dynamics of DNA repair proteins *in vivo* which is at present also achievable using gridded USX [3] as a source of low LET IR. However, careful consideration should be given to the laser microbeam system used, the types of DNA damage induced at the laser wavelength chosen and the density of the damage, which relates to the average peak power, photon flux and laser dwell time used during irradiation, as previous experimental data are at times contradictory with regard to protein kinetics at sites of damage. Additionally, damage induced following laser microbeam irradiation may need to be considered as different substrates based on the structural and chemical complexity of the damage sites containing two or more lesions in close proximity, particularly as the persistence of damage corresponds with the types of complex damage induced by high LET radiation. Research using laser microbeams, whilst a relative new tool, is making rapid progress in furthering our understanding of the repair of complex DNA damage that poses a significant threat to cellular integrity. Laser micro-irradiation may be very useful to shed light on the repair of replication induced DSBs formed when complex lesions progress through to replication causing stalled replication forks, which can lead to chromosomal translocations [64] or mutations [65] and are seen as potential targets for synthetic lethality.

## Conflict of interest statement

None declared.

## Funding source

Science and Technology Facilities Council Biomed Network [HNB3003] and Medical Research Council [MC_PC_12001].

## Figures and Tables

**Table 1:** Qualitative comparison of the spectrum of lesions induced following IR and laser microbeam irradiation.

Treatment	IR low LET	UV laser	Heavy ion beam	NIR laser	
				−	+
				Photosensitizer	
Base lesions	+++	+	++	+	+++
CPDs and 6,4-phototproducts	−	+++	−	+	−
SSBs	++	+	+	+/−	++
DSBs					
Direct	+	+/−	+	+/−	+
Indirect	+	+/−	+	+/−	+
